# Education Research: Moving Beyond “Outstanding”: Neurology Program Directors' Perspectives on the MSPE Clerkship Summaries

**DOI:** 10.1212/NE9.0000000000200319

**Published:** 2026-06-11

**Authors:** Robert Thompson Stone, Tamara B. Kaplan, Stephen Joseph Powell, Catherine Nicholas, Samantha P. Myers, Melanie Braun, Andres Fernandez, Christopher J. Mooney

**Affiliations:** 1 Department of Neurology, University of Rochester, NY;; 2 Department of Neurology, Harvard University, Boston, MA;; 3 University of Rochester School of Medicine and Dentistry, NY;; 4 Department of Neurology, Thomas Jefferson University, Philadephia, PA; and; 5 Departments of Medicine, Health Humanities and Bioethics, and Public Health Sciences, University of Rochester, NY.

## Abstract

**Background and Objectives:**

As standardized metrics in residency selection change, residency program directors increasingly rely on narrative components of the Medical Student Performance Evaluation (MSPE) clerkship summaries. However, wide variability in structure, language, and transparency limits their usefulness for high-stakes decision making. The aims of this study were to explore neurology and child neurology program directors' perspectives on the utility of MSPE neurology clerkship summaries and to identify features that enhance or undermine their value in residency selection.

**Methods:**

We conducted a qualitative study using semistructured interviews with US neurology and child neurology residency program directors. Participants reviewed and discussed 4 constructed MSPE neurology clerkship summary cases developed from deidentified authentic MSPE excerpts and designed to reflect variation in structure and content. Interviews were audio recorded, transcribed verbatim, and analyzed using reflexive thematic analysis informed by theories of narrative assessment and discourse. Trustworthiness was supported through analytic memos, peer debriefing, and investigator triangulation.

**Results:**

Thirteen program directors participated, and 6 themes emerged: (1) Participants valued honest and transparent narratives but perceived candid discussion of limitations as risky for students; (2) institutional variability in grading systems, access to neurology experiences, and evaluator practices limited fair comparison of applicants; (3) narratives were most useful when clearly structured, concise, and transparent about how evaluations were synthesized; (4) comparative information was viewed as essential for decision making, despite its susceptibility to bias; (5) program directors consistently reported that coded language was difficult to interpret and should be avoided; and (6) nonspecific laudatory comments diminished narrative value, whereas brief, concrete behavioral examples enhanced credibility and interpretability.

**Discussion:**

Neurology clerkship summaries in the MSPE remain influential yet imperfect tools for residency selection. Program directors favor narratives that are structured and specific and avoid coded or vague language. Targeted faculty development and adoption of shared narrative frameworks represent actionable strategies to improve fairness, clarity, and trust in narrative assessment within neurology education.

## Introduction

Residency selection has become an increasingly complex decision-making process, marked by growing uncertainty and diminishing availability of standardized metrics. As programs navigate this evolving landscape, understanding how narrative evaluations such as the Medical Student Performance Evaluation (MSPE) clerkship summary are used, and how they might be improved, is essential. The 2022 transition of the United States Medical Licensing Examination (USMLE) Step 1 to pass-fail^1^ intensified uncertainty about how programs would compare and select applicants.^2,3^ Other numerical assessments including the USMLE Step 2 Clinical Skills examination, clerkship grades, and class rankings have also been de-emphasized or eliminated. With fewer standardized metrics, residency program directors are relying more on qualitative application elements.

The clerkship summary, which we define as the section of the MSPE describing a student's clerkship performance, typically includes grading distribution, final grade, and narrative comments. However, the value of these summaries is limited by inconsistency, lack of clarity, and variability in standardization. The credibility of narrative comments is further undermined by linguistic variability or inconsistent language use to describe student performance. Such variation includes adjective choice and meaning (e.g., use of keywords), as well as differences in narrative length, specificity, and domains addressed.^4^ Together, these variations limit comparability and can introduce bias into interpretation.^5^ Multiple studies have identified this variability as a major barrier to the MSPE's usefulness.^6-9^

For the MSPE clerkship summary to communicate effectively, it must convey truthfulness, specificity, relevance, and structure.^10^ Yet the summaries often fall short, leading program directors to question their credibility.^7,8,11^ Some specialties have responded to these challenges by developing alternative approaches. The Council of Emergency Medicine Residency Directors, for example, created the standardized letter of evaluation, which includes a comparative global assessment and structured 250-word narrative addressing noncognitive attributes.

Despite extensive critique of the MSPE and clerkship summaries, less is known about how program directors perceive their usefulness in the current residency selection landscape and how clerkship directors and departments can improve their value, particularly in neurology. This study addresses this gap by qualitatively examining neurology program directors' perspectives on the utility of MSPE neurology clerkship summaries using in-depth semistructured interviews. We used constructed case summaries as vignette-based prompts to elicit participants' reflection and decision-making processes. These materials functioned as discussion stimuli rather than as evaluative instruments or proposed templates. This approach allowed us to capture program directors' interpretive frameworks and reasoning in context, rather than relying on survey responses or indirect inferences about their practices. Through this work, we sought to identify factors that enhance or undermine the usefulness of clerkship summaries in guiding interview and ranking decisions. Our findings are intended to inform practical recommendations for neurology clerkship directors, departments, and national stakeholders to better support program directors' decision making.

## Methods

### Design

We performed a qualitative study informed by a constructivist paradigm, using iterative semistructured interviews and reflexive thematic analysis.^12^ This approach enabled systematic exploration of how neurology and child neurology program directors interpret and evaluate neurology clerkship summaries in the MSPE.

### Development of MSPE Case Summaries and Interview Script

We developed 4 example MSPE neurology clerkship summaries (“case summaries”) representing the range and variability observed in actual MSPEs. One author (R.T.S.) created them using deidentified excerpts from MSPEs submitted during the 2022–2023 application cycle. To ensure meaningful variation, we convened a multidisciplinary group including 2 clerkship directors, a child neurology program director, a fourth-year medical student, 2 neurology residents, and a director of assessment. Through discussion, the group identified key attributes influencing MSPE narratives, including specificity, data transparency, narrative length, formatting, comparative descriptors, attention to areas for improvement, and keyword use. Iterative feedback guided revisions until consensus was reached on 4 final case summaries (e.g., changes were made to include summaries with and without grade distributions). Two members of the research team (R.T.S., T.B.K.) developed a semistructured interview guide using these summaries as vignette-based prompts to elicit participants' reflections, interpretative frameworks, and prior experiences, rather than to solicit bounded evaluations of the documents themselves. We piloted the guide with 1 program director and made minor refinements before data collection (e.g., reducing redundant prompts and adding a question about the potential for bias in student narratives).

### Participant Recruitment and Data Collection

We recruited US-based neurology and child neurology residency program directors using a publicly available national directory, announcements on an American Academy of Neurology online forum, and personalized email invitations within the authors' networks. All participants received a study information letter. Using maximum variation sampling, we purposively selected respondents to reflect diversity in program size, type, and geographic region. Program size was categorized as small (<8 residents in neurology; <3 in child neurology), medium (8–16; 3–4), and large (>16; >4). All program directors represented university-based programs. Enrollment and analysis occurred iteratively in small cohorts (typically 3 participants), allowing concurrent data collection, coding, and refinement of interview prompts.

Three coinvestigators (T.B.K., R.T.S., M.B.) conducted all Zoom interviews. Interviews were audio recorded and transcribed verbatim using Temi.com.

### Analysis

We conducted a reflexive thematic analysis following Braun and Clarke's 6-phase framework, using a theory-informed interpretive approach.^13^ Analysis drew on theories of narrative assessment and evaluative discourse, which conceptualize assessment as an interpretive process shaped by evaluators' experiences, assumptions, and professional norms.^14-16^ These ideas informed interview design and analysis: For example, the guide included prompts about words and phrase interpretation, and analysis examined how linguistic variability shaped decision making.

We also drew on scholarship addressing the quality and interpretability of narrative assessment, emphasizing clarity and specificity in conveying meaningful judgments about performance.^17,18^ Accordingly, we examined how participants evaluated the usefulness and interpretability of MSPE narratives varying in linguistic precision and transparency, for example, by asking participants to elaborate on aspects of the summaries that were vague or were open to interpretation. These theories served as sensitizing concepts to deepen the understanding of how program directors interpreted MSPE case summaries.

Four team members (R.T.S., S.M., S.P., C.N.) conducted in-depth readings of transcripts to develop familiarity. To initiate coding, the team jointly analyzed one transcript and developed a preliminary codebook. We then worked in dyads to analyze remaining transcripts: One member performed line-by-line inductive coding,^19^ while the other reviewed and refined it; pairs then met to reach consensus. We applied a latent thematic approach^12^ to organize codes into candidate themes and subthemes. This recursive process ensured themes were conceptually sound, data supported, and representative of participants' perspectives.^20^ In the final phase, the full study team met to refine themes, resolve discrepancies, and clarify thematic boundaries. We used Dedoose (v.9.0.17, SocioCultural Research Consultants, LLC) for data organization and analysis.

We applied strategies aligned with Lincoln and Guba's criteria to ensure trustworthiness.^21^ We supported credibility through analytic memoing, peer debriefing, investigator triangulation, and member checking. During interviews, the interviewers periodically paraphrased and summarized participants' statements to confirm accuracy and meaning. During manuscript revision, we shared a written summary of themes with participants and invited feedback; respondents affirmed the findings without substantive concerns. We supported dependability through an audit trail and confirmability by grounding interpretations in participant accounts and reflexive dialog. We prioritized authenticity by representing participants' voices and transferability through detailed descriptions of the educational setting, sampling strategy, and study procedures.

### Reflexivity

Our team brought diverse perspectives that shaped the study design, analysis, and interpretation of results. The team included 3 neurologist educators, 1 education scientist, and 3 trainees. R. Thompson Stone is a neurology clerkship director and child neurology residency program director with expertise in narrative assessment. T.B. Kaplan is a neurology clerkship director and vice-chair of education with experience in medical school leadership and assessment. C.J. Mooney is a qualitative education scientist. M. Braun is an associate neurology clerkship director. At the time of the study, SP was a neurology resident, C. Nicholas was a child neurology resident, and S.P. Myers was a 4th-year medical student applying to child neurology.

Throughout the study, we engaged in reflexive dialog to examine how our roles, positionalities, and underlying assumptions shaped the research process. For example, clinician-researchers' familiarity with the MSPE contextualized participants’ remarks, while CM's outsider perspective challenged assumptions. These complementary perspectives enhanced analytic rigor and depth.

### Standard Protocol Approvals, Registrations, and Patient Consents

This study (STUDY00008663) was approved by the University of Rochester Medical Center's Research Subjects Review Board, which waived the requirement for written informed consent.

### Data Availability

Anonymized data not published within this article will be made available by request from any qualified investigator.

## Results

Thirteen program directors participated in the interviews (Table 1), and we included our pilot interview in the thematic analysis. We generated 6 key themes regarding perceptions of the neurology clerkship grade summaries in the MSPE. The themes and key ideas are summarized in Table 2. Representative excerpts from the sample MSPE case summaries, alongside illustrative participant quotes, are listed in Supplemental Table 1, demonstrating how the summaries prompted discussion of each identified theme.

**Table 1:** Characteristics of Residency Programs Represented by Participating Program Directors (n = 13)

Program characteristics		
	n	%
Specialty		
Adult Neurology	9	69.2
Child Neurology	4	30.8
Geographic Region		
Midwest	2	15.4
Northeast	5	38.4
Southeast	2	15.4
Southwest	2	15.4
West	2	15.4
Size		
Small	4	30.8
Medium	6	46.1
Large	3	23.1

**Table 2:** Main Themes and Key Insights

Main theme	Key insights
Greater honesty and transparency in MSPE narratives is needed, though it carries increased risk for the student	• Program directors distrust overly curated narratives and prefer candid reporting • Disclosure of limitations and areas for improvement will likely disadvantage students in a competitive match environment
Fair and reliable judgments are limited by institutional variability	• Student access to neurology experiences varies, disadvantaging those with fewer opportunities • Variability in evaluative structures and MSPE formats across schools hinders fair comparison of applicants • Narrative strength may reflect evaluator advocacy and skill more than student ability
Narratives are most useful when clearly structured, streamlined, and transparent about how evaluations are synthesized	• Narratives are most easily interpreted when organized around defined competencies or performance domains • Narratives should balance length—neither too long as to be overwhelming or redundant nor too short as to be uninformative • Clarity in identifying the role of evaluators is helpful when interpreting comments • The content of grades and narrative evaluations can be contradictory, which is difficult to interpret
Comparative information is critical for decision making, but prone to problematic elements	• Comparative data are required for differentiation between students • Minimizing or eliminating one type of comparator necessitates the use of others, each with their own limitations • Loss of quantitative metrics increases reliance on strong narrative comments for fair assessment • Comparative data introduces potential biases, but these might be mitigated by balanced, high-quality summaries
Coded language (i.e., keywords) is hard to interpret, creates confusion, and should be avoided	• Keyword meaning varies widely across institutions and evaluators, creating uncertainty in narrative interpretation • Explicit faculty training to avoid coded language may reduce misinterpretation
Abundant laudatory comments lacking specificity lead to loss of meaning, reducing the usefulness of the MSPE narrative	• Specific behavioral examples mitigate the loss of meaning associated with vague or uniformly positive praise by providing context and clarity • Distrust of uniformly positive narratives leads program directors to screen for negative comments as red flags

Abbreviation: MSPE = Medical School Performance Evaluation.

### Greater Honesty and Transparency in MSPE Narratives Is Needed, Though It Carries Increased Risk for the Student

Program directors endorsed the value of honest and transparent reporting in MSPE clerkship summaries and expressed frustration about the selective presentation of comments. Many perceived that comments were curated to portray students in the best possible light, “buffed up, glossed over, glamor shots of a student” (P14), while areas for growth were “often hidden” (P3), limiting the MSPE's usefulness in selection decisions:

There’s a way for everyone to get very good comments, and it’s very hard to identify students who are performing poorly. (P7)

However, participants emphasized that candid discussion of growth areas was rare and perceived as “too risky to implement” (P3) in a competitive match environment and a “huge red flag” (P7):

I was actually shocked to see areas for growth because I think almost every single MSPE leaves that out. I feel torn about whether that should be included in an MSPE…you just don’t want to ever have that hurt [the student]. (P1)

Despite these risks, some supported developing a “standardized recommendation” (P5) for including constructive feedback, suggesting it could enhance fairness and help residency programs better support new trainees:

That would be great because then you understand the opportunities for students to develop during residency and … where that student might struggle. (P5)

### Fair and Reliable Judgments Are Limited by Institutional Variability

Program directors emphasized that the utility of the MSPE is constrained by wide inconsistencies in access to neurology opportunities, narrative content, and grading systems, which undermines fair comparison among applicants.

Given the “variability in neurology departments around the country and how neurology is taught” (P14), some students have fewer opportunities, particularly those without a required neurology rotation. Program directors were concerned about “incorrectly penalizing” (P2) students who “really have to fight to get some neuro exposure” (P2):

If you're at a large institution with lots of neurologists and a core clerkship and then a sub-I after that, [you have] eight weeks to really show yourself. [But], if you're at a community-based center, you [only have the opportunity to] do an elective. (P9)

In addition, substantial variation in the MSPE clerkship summaries, ranging from highly detailed and domain specific to vague and difficult to interpret complicated efforts to “compare apples to apples” (P14), particularly as “a lot of schools [are] going pass/fail and [others] not” (P2):

There’s a lot of inconsistency across schools, which makes it very hard. Some of them are much more specific. Some of them have grades, some of them have more language to help you understand the MSPE and what you’re reading and how to use it. Others… they just throw it at you. (P2)

Many program directors worried that evaluator differences, such as writing ability, engagement, and advocacy, could overshadow actual student performance. They felt the content could reflect “who is writing them and not so much necessarily on the applicants” (P8), while others acknowledged that “an endorsement statement from one neurologist to another” (P11) could powerfully shape interpretation:

You're penalizing a student who went to a school where the [evaluators]…write a short paragraph…that might not reflect the student's performance at all. That might be the evaluator issue. So that’s a struggle, you want to reward students [with strong evaluations], but are you just rewarding them because they have better evaluators? (P7)

### Narratives Are Most Useful When Clearly Structured, Streamlined, and Transparent About How Evaluations Are Synthesized

Program directors preferred narratives organized around defined competencies or performance domains rather than unstructured paragraphs and valued narratives that were concise and nonredundant while still providing sufficient substantive detail:

Most program directors who are reviewing applications really try and look through the whole application. When you do that and your eyes are just kind of spinning in your head, seeing a wall of text, it makes it more likely for me to be skipping over things if I've been at it for four hours. (P3)

Participants also wanted clarity in evaluator roles, noting that identifying the source of each comment, such as “knowing attendings versus residents” (P3) provided valuable interpretive context, and would help reduce “assumptions about who the narrator might be” (P3):

The overall summary paragraph from the clerkship director… takes all the comments together and puts them in one paragraph. You don’t have quantitative proof that this student worked with a number of different people. (P7)

However, participants described challenges interpreting discrepant evaluator comments, and there was no consensus on whether it was the clerkship director's role to reconcile or preserve differing information:

Do [clerkship directors] have free reign to leave [comments] out because in aggregate it looks like this person is so outstanding and this outlier just can be left out? Or do they try to just put in every flavor that comes through? (P10)

### Comparative Information Is Critical for Decision Making But Is Prone to Problematic Elements

Program directors consistently emphasized that comparative data, such as grades or narrative comparators, are essential for differentiating students and guiding interview and ranking decisions. Without “something that compares the student to a standard” (P11), they found that the information was insufficient to interpret performance and led to further homogeneity of the application:

I feel like everyone has become homogenous really in a lot of senses. Like every single letter of recommendation says the same thing. Everyone does the exact same community service. And so, I really struggle, it's hard when you take out anything that's in any way trying to distinguish applicants. (P4)

At the same time, program directors were frustrated by inflated or homogenized distributions as “giving grade distributions without meaningful distinction between groups is not helpful.” (P11)

To improve equity, changes have been made to reduce or eliminate comparative data that may be inherently biased (e.g., clerkship grades). Program directors acknowledged these efforts as important but noted that removing one comparator does not eliminate the need to differentiate applicants. Inevitably, they turn to alternative comparators (e.g., school reputation), which “of course also have biases.” (P5)

In particular, participants report that “it is difficult to know how to compare students who are coming from an ‘honors’ school with students coming from a ‘pass/fail’ school” (P7). As one noted, “the bar is a lot higher for schools that don't use grades to be really structured and clear about how they are evaluating students” (P10). In this context, detailed, descriptive, and differentiating narratives become critical, as one program director explained, if they are “not convinced that someone is clinically strong and ready for residency, why take the chance?” (P10)

Does this student get rewarded because they went to a school with honors and the other student could have gotten honors but did not because the school is pass/fail? Does [the latter] student get penalized when they are compared? (P7)

Despite their need for comparative data, program directors acknowledged that such information is susceptible to inconsistency and bias, often in ways that are difficult for program directors to detect and address:

There’s inherent bias in the grading structure and the evaluations and the compiling of evaluations… some may bias towards those who are more outgoing. And then obviously there are factors that I would not have enough information to know whether other student characteristics and identity would've played a part such as gender or ethnicity or where they went to college. So yes, there's definitely a 100% chance that bias was introduced in both the raw data and in the composing of it. (P14)

Program directors suggest that the impact of bias could be reduced when multiple, high-quality, and well-structured data sources, balanced across quantitative and qualitative data, are available:

I think having more data in forms of assessment is helpful, because if you rely on one [that] has a particular bias, then you're probably moving too far in one direction. If you have more [forms of assessment], then hopefully people [are more able to do] a holistic review. (P5)

### Coded Language (i.e., Keywords) Is Hard to Interpret, Creates Confusion, and Should Be Avoided

Most program directors consistently noted the frequent use of “coded language,” including keywords such as “outstanding,” “excellent,” and “very good”, whose meanings vary widely across institutions and evaluators. This variability leaves readers uncertain about “how to interpret” (P1) the language, which was described as “empty and vague” (P1)

There’s a lot of use of qualitative words, like good and excellent, that make it seem like there’s an implicit coding going on that I’m not aware of. [For example, saying] ‘she was an outstanding student’, ‘good write-ups’, ‘excellent exam of patients’, ‘good attention to detail’,‘good relationships with patients and stroke team’: So does that mean she is actually an outstanding student that excels at all these things? Or is it actually trying to tell me that while she’s good at exams, [she is] actually average or maybe even less than average on all these other things? Because as we all know, the word good is one of the worst things that you can say. (P10)

Program directors also expressed concern that many evaluators may be unaware of the implicit “codes” embedded in their narratives, inadvertently disadvantaging students by using terms that carry unintended meanings:

I don’t know if people are using code words or not, but unless it’s explicitly stated… somebody might not even know that there is such a thing as code words. Maybe this writer could say something, write something that’s detrimental to a student without really meaning to. (P11)

Ultimately, most program directors felt that coded language should be avoided, recommending that “they train the clerkship directors not to use codes in their narratives” (P11) and that schools explicitly state when coded language is absent.

I would personally find [it] really helpful…to get rid of the coded language and different interpretations [of that] language. (P4)

### Abundant Laudatory Comments Lacking Specificity Lead to Loss of Meaning, Reducing the Usefulness of the MSPE Narrative

Program directors expressed frustration with the overabundance of laudatory language in narratives, which makes them “less and less meaningful” (P13) as “there are only so many superlatives you can add onto ‘outstanding’” (P5). They emphasized that including specific examples can help mitigate this loss of meaning by providing supportive evidence as “it provides a little bit more meat to the generalized statements” (P13).

They did give some specific examples, [for example], ‘able to name congenital aqueduct stenosis in the differential diagnosis’. I liked that they put something [specific like] that and didn't just put this random ‘highly recommended’. She's acting at the level of PGY-1 and these are the things that she said or did that supported that statement. (P2)

There are a lot of very specific comments about performance, [for example], ‘tailoring the neuro exam with the HINTS [exam]’. And then ‘handling the pressure in difficult cases, specifically [a] patient with non-accidental trauma’. There are so many specific examples of things that this student did well, that it really gives you a concrete idea of how they might perform as a resident. (P7)

Because nearly all MSPE narratives are overwhelmingly positive, some program directors described reading between the lines and “screening more for something concerning” (P12) such as any negative comments, reflecting a lack of trust in uniformly positive narratives:

I'm really looking more for what's omitted. And if I see a low performance, try to figure out why that was, because it's never stated, it's never stated why the student did so poorly. And so I have to look for what is omitted and try to make inferences. (P11)

## Discussion

As traditional selection metrics decline or disappear, program directors increasingly rely on narrative assessments to inform high-stakes residency decisions. Our findings highlight both the challenges and the opportunities for strengthening the MSPE neurology clerkship summary and suggest actionable strategies to enhance the clarity, fairness, and interpretability.

A central tension identified in our study was the balance between transparency and student advocacy. Program directors consistently expressed a desire for honest descriptions of performance yet acknowledged that candid discussion of limitations or areas for growth would disadvantage students in a competitive match environment. Many studies cite a lack of transparency in the MSPE, stemming from a tension between medical schools' desire to present their students favorably and program directors' need for balanced, candid assessments.^5,6,9,22-24^ As a result, clerkship narratives tend to omit negative comments and lack specific details on professionalism concerns.^22^ Unsurprisingly, there was no consensus on whether including areas for improvement should be standard practice, reflecting the ethical and practical challenges of transparency in high-stakes assessment.

Program directors also emphasized transparency in how multiple evaluators' perspectives are synthesized. Clear attributions of comments to evaluator role (e.g., resident versus attending) provides context, particularly with discrepant interpretations. Clerkship directors must therefore decide whether to present divergent perspectives or to integrate them into a unified representation of performance.

Consistent structure further enhanced MSPE utility. Program directors preferred summaries organized around defined competency or performance domains rather than unstructured text. Formatting features such as bullet points and domain headings improved readability and supported efficient application review. Attention to length was equally important because redundancy impeded review, while overly brief summaries lacked substance. A consistent, predictable structure reduces extraneous cognitive load and supports efficient information retrieval, a priority aligned with previous studies and the AAMC MSPE Task Force.^25,26^

Our findings underscore that MSPE clerkship narratives do not exist in a vacuum; they are shaped by institutional context, evaluator skills, and access to neurology experiences. Prior work has documented wide variability in evaluators' ability to provide specific, behaviorally anchored comments,^16,27^ and participants similarly noted that narrative quality often reflects evaluator advocacy as much as, or sometimes more than, student abilities. Although faculty development can improve narrative quality, such efforts are often voluntary and disproportionately engage already skilled evaluators,^28,29^ allowing variability to persist.

Inequities are compounded for students with limited access to neurology experiences or mentorship. As of 2017–2018, clinical neurology was required in 86% of allopathic and 6% of osteopathic medical schools,^30,31^ resulting in substantial differences in opportunities to generate meaningful narrative evidence. While direct links between neurology exposure and MSPE quality are limited, mentorship within a chosen specialty is known to influence match outcome.^32^ These findings underscore the need for national advocacy and expansion of student mentorship programs, particularly for students at schools with limited access to neurology.

While variability across institutions is unlikely to be eliminated, program directors demonstrate awareness of these inequities when reviewing applications, which is an initial step. Development of nationally shared frameworks, exemplars, and resources could help level the playing field, ensuring greater consistency across institutions.

Program directors consistently emphasized the importance of comparative data for differentiating applicants, such as grade distributions, class rank, and comparative comments (e.g., “performed at the level of a first-year resident”). As traditional metrics such as Step 1 and clerkship grading shifts to pass/fail, programs increasingly rely on narrative assessments.^2,33^ In one scoping review, 96% of program directors reported that pass/fail grading made objective comparison more difficult and anticipated greater reliance on narrative summaries.^34^

In this context, small wording differences function as implicit comparisons, with individual adjectives carrying disproportionate weight. This aligns with prior work demonstrating faculty concern about the consequences of their narrative comments being used for high-stakes decisions, particularly as linguistic patterns may reflect systemic and implicit racial and gender biases.^35-37^ Despite these limitations and inconsistent benchmarks,^38,39^ few studies have addressed how program directors reconcile these concerns with their need to compare applicants. Our findings suggest that balanced integration of quantitative and qualitative information, presented with clarity and context, is most useful.

Specificity emerged as fundamental to narrative utility and trustworthiness. Program directors expressed near-universal frustration with coded terms such as “outstanding” or “excellent,” whose meanings vary widely across institutions and evaluators. Similarly, the overabundance of generic, laudatory language rendered students indistinguishable and diminished the narrative's value. By contrast, brief exemplars of observed behaviors or clinical reasoning provided meaningful insight into performance, making specificity the most credible form of comparison, grounded in evidence rather than abstract rankings.

Faculty development should therefore prioritize specificity, training evaluators to anchor comments in directly observed behaviors and skills. Embedding prompts, exemplars, and reminders into evaluation forms could discourage keywords and promote concrete examples. Prior work in neurology using the Narrative Evaluation Quality Instrument demonstrates that targeted faculty development and systemic changes can improve narrative specificity.^17^ Promoting specificity represents a tangible and achievable step toward improving fairness in residency selection, and broad implementation of such strategies could support continuous quality improvement.

This study has limitations. Case summaries could not capture the full range of MSPE formats, so some discussion points may be unrepresented. Findings reflect perceptions rather than observed selection decisions, leaving open the possibility that reported practices differ from real-world behavior. However, using authentic case summaries allowed participants to engage with realistic narrative language rather than abstract descriptions of their practices. Although we analyzed perspectives on quantitative data (e.g., themes 1, 2, and 4), the analysis focused primarily on narrative comments (e.g., themes 3, 5, and 6), which we viewed as the MSPE section most directly shaped by neurology faculty. Consequently, themes related to quantitative data collection and presentation may be less developed. Our sample included program directors from a limited number of institutions and only university-based programs, so perspectives from community-based program directors are not represented. However, consistent themes across programs of varying sizes, geography, and types suggest broader relevance.

MSPE clerkship summaries remain a critical yet imperfect tool in residency selection, and meaningful improvements will require efforts by clerkship directors, departments, institutions, and national stakeholders. We provide recommendations for all levels (Table 3) linked with rationale related to our themes. Ultimately, the MSPE's value lies not in uniform positivity but in disciplined specificity—narratives that earn trust through evidence, context, and clarity. Achieving this will require coordinated institutional and national efforts to support evaluator training, standardized structures, and ethical representation of student performance. Without such collective action, the MSPE risks remaining a curated sales pitch rather than a credible assessment of readiness for residency.

**Table 3:** Recommendations for Improving the MSPE Neurology Clerkship Summary

Stakeholder	Recommendation	Rationale
Clerkship Directors	Organize clerkship summary comments by competency or domain	Improves readability and efficiency of review (theme 3)
	Balance length of comments to be efficient and nonredundant, yet thorough (e.g., 150–200 words)	Balances depth with clarity; avoids overwhelming PDs (theme 3)
	Include “synthesis statements” clarifying how evaluations were reconciled, including identifying evaluator roles	Provides more transparent, coherent information (themes 1 and 3)
	Preferentially include comments with specificity and comparators, avoiding coded and vague language	Reduces ambiguity and loss of meaning (themes 4, 5 and 6)
	Use clerkship evaluation forms with prompts to elicit concrete examples and avoid coded language	Enhances comment specificity and reduces ambiguity (themes 5 and 6)
Departments	Promote faculty training for evaluators to improve specificity, particularly those who may not otherwise participate	Expands skills of faculty leading to improved specificity (themes 2, 5 and 6)
	Ensure the presence of a strong departmental mentor team, including for visiting students without access to mentors	Increases fairness in comparison across schools (theme 2)
National Stakeholders	Advocate for national standards in MSPE Neurology Clerkship summary structure and transparency	Mitigates inequities across institutions, standardizes useful structural elements (themes 1, 2, and 3)
	Expand national mentorship programs to enhance equitable access to neurology experiences	Addresses variability in student exposure and access (theme 2)
	Develop and disseminate tools to assess narrative quality	Provides resources for departments to work on continuous improvement and align with national practices (themes 2, 4, 5, and 6)

Abbreviation: MSPE = Medical School Performance Evaluation.

## Glossary

MSPE: Medical Student Performance Evaluation
USMLE: United States Medical Licensing Examination
